# Supplementary material for: Synthetic mRNAs Containing Minimalistic Untranslated Regions Are Highly Functional In Vitro and In Vivo
Source: Cells. 2024 Jul 24;13(15):1242. doi: 10.3390/cells13151242 (PMC11311775; doi:10.3390/cells13151242)
Supplement: Supplementary file 1 [file cells-13-01242-s001.zip › cells-3041220-supplementary.pdf]

SUPPLEMENTARY DATA

Figure S1

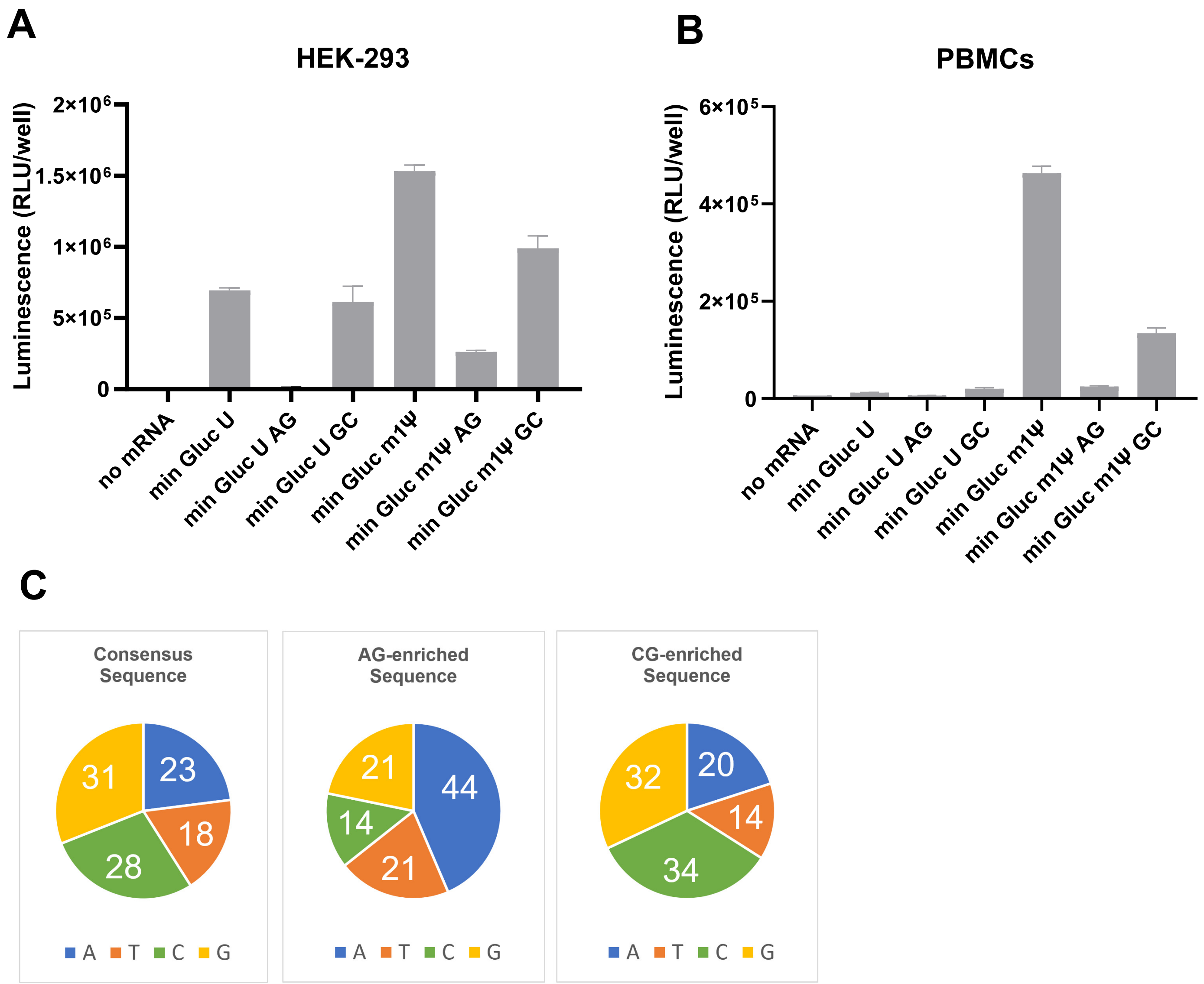
A
HEK-293
Luminescence (RLU/well)
2×10^6
1.5×10^6
1×10^6
5×10^5
0
no mRNA
min Gluc U
min Gluc U AG
min Gluc U GC
min Gluc m1Ψ
min Gluc m1Ψ AG
min Gluc m1Ψ GC
B
PBMCs
Luminescence (RLU/well)
6×10^5
4×10^5
2×10^5
0
no mRNA
min Gluc U
min Gluc U AG
min Gluc U GC
min Gluc m1Ψ
min Gluc m1Ψ AG
min Gluc m1Ψ GC
C
Consensus Sequence
23
18
28
31
A T C G
AG-enriched Sequence
44
21
14
21
A T C G
CG-enriched Sequence
20
14
34
32
A T C G

Figure S2

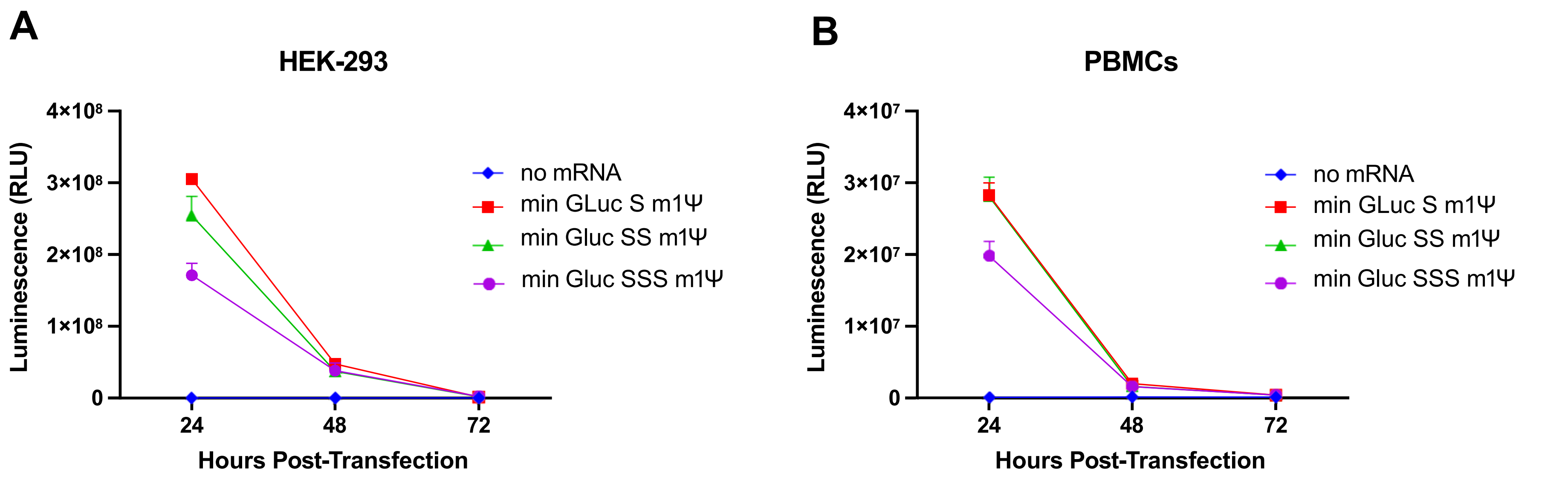
A
HEK-293
4×10⁸
3×10⁸
2×10⁸
1×10⁸
0
Luminescence (RLU)
24
48
72
Hours Post-Transfection
no mRNA
min GLuc S m1Ψ
min Gluc SS m1Ψ
min Gluc SSS m1Ψ
B
PBMCs
4×10⁷
3×10⁷
2×10⁷
1×10⁷
0
Luminescence (RLU)
24
48
72
Hours Post-Transfection
no mRNA
min GLuc S m1Ψ
min Gluc SS m1Ψ
min Gluc SSS m1Ψ

Figure S3

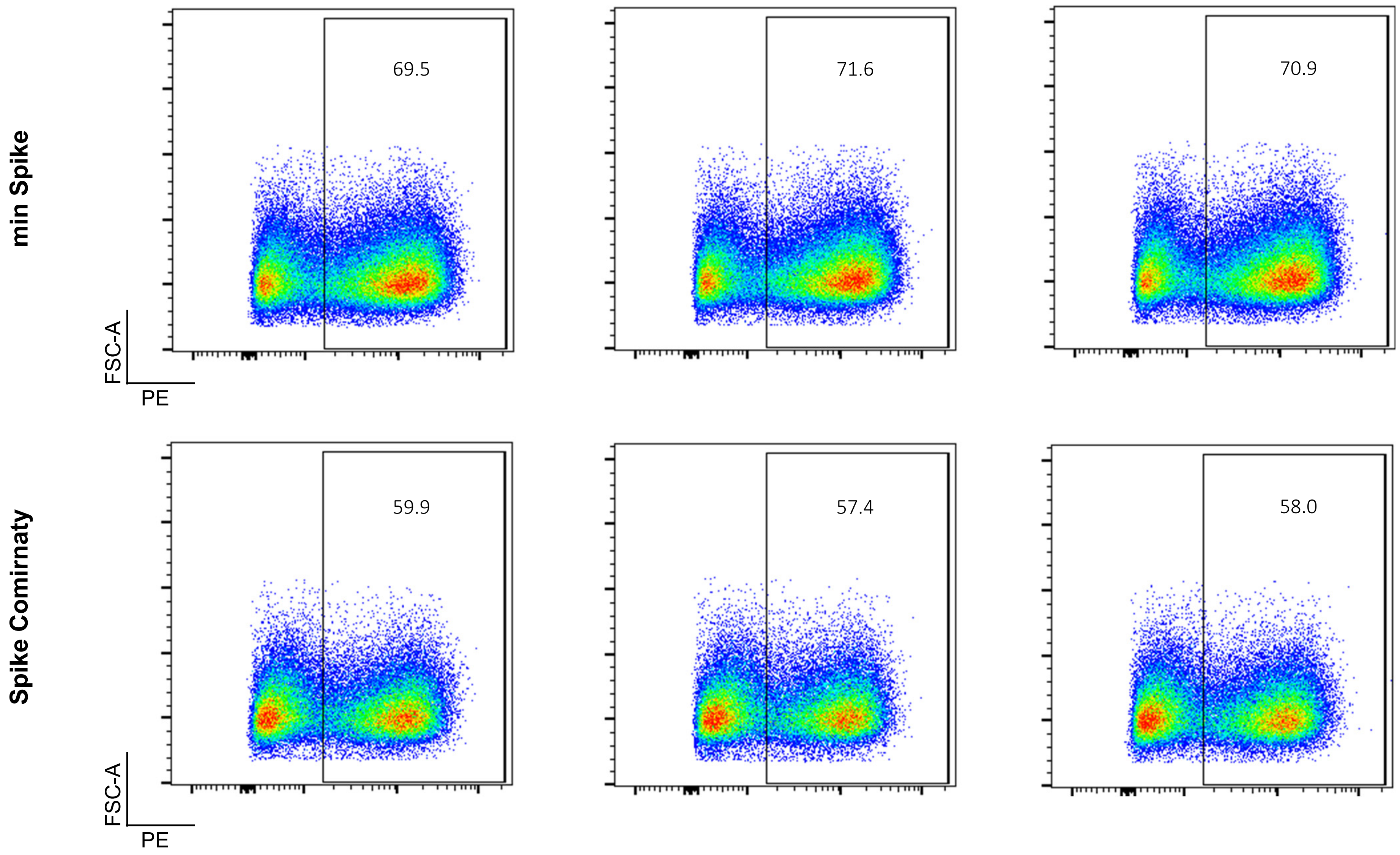
min Spike
Spike Comirnaty
69.5
71.6
70.9
59.9
57.4
58.0
FSC-A
PE
FSC-A
PE

Table S1

mRNA	
	**5' UTR**
**no 5'-UTR**	mGpppAmG<u>AUG</u>
**Kozak**	mGpppAmGACC<u>AUG</u>
**TISU**	mGpppAmGCAAG<u>AUG</u>
**opt**	mGpppAmGACUCACUAUUUGUUUUCGCGCCCAGUUGCAAAAAGUGUCGUCACC<u>AUG</u>
**Spikevax**	mGpppAmGAAAUAAGAGAGAAAAGAAGAGUAAGAAGAAAUAUAAGACCCCGGCGCCGCCACC<u>AUG</u>
	**3' UTR**
**bgbg**	GAGAGCUCGCUUUCUUGCUGUCCAAUUUCUAUUAAAGGUUCCUUUGUUCCCUAAGUCCAACUACUAAACUGGGGGAUAUU<br>AUGAAGGGCCUUGAGCAUCUGGAUUCUGCCUAAUAAAAAACAUUUAUUUUCAUUGCUGCGUCGAGAGCUCGCUUUCUUGC<br>UGUCCAAUUUCUAUUAAAGGUUCCUUUGUUCCCUAAGUCCAACUACUAAACUGGGGGAUAUUAUGAAGGGCCUUGAGCAU<br>CUGGAUUCUGCCUAAUAAAAAACAUUUAUUUUCAUUGCUGCGUCGUCGACAAUCAACCUCUGGAUUACA<u>nA</u>
**mtRNR1-AES**	CUCGAGCUGGUACUGCAUGCACGCAAUGCUAGCUGCCCCUUUCCCGUCCUGGGUACCCCGAGUCUCCCCCGACCUCGGG<br>UCCCAGGUAUGCUCCCACCUCCACCUGCCCCACUCACCACCUCUGCUAGUUCCAGACACCUCCCAAGCACGCAGCAAUGC<br>AGCUCAAAACGCUUAGCCUAGCCACACCCCCACGGGAAACAGCAGUGAUUAACCUUUAGCAAUAAACGAAAGUUUAACUAA<br>GCUAUACUAACCCCAGGGUUGGUCAAUUUCGUGCCAGCCACACCCUGGAGCUAGCAA<u>nA</u>
**Spikevax**	GCUGGAGCCUCGGUGGCCUAGCUUCUUGCCCCUUGGGCCUCCCCCCAGCCCCUCCUCCCCUUCCUGCACCCGUACCCCC<br>GUGGUCUUUGAAUAAAGUCUGAGUGGGCGGCA<u>nA</u>
